# Sounds That People with Visual Impairment Want to Experience

**DOI:** 10.3390/ijerph18052630

**Published:** 2021-03-05

**Authors:** Rafal Mlynski, Emil Kozlowski, Jan Adamczyk

**Affiliations:** Department of Vibroacoustic Hazards, Central Institute for Labour Protection—National Research Institute, Czerniakowska 16, 00-701 Warsaw, Poland; emkoz@ciop.pl (E.K.); adamczyk@ciop.pl (J.A.)

**Keywords:** sound, visual disability, blindness, visual impairment, noise

## Abstract

This article presents the expectations of visually impaired people with regard to the content of a set of sound exercises planned for implementation, which will mainly enable these people to become familiar with the sounds associated with specific life situations. Consultations were carried out with 20 people with visual impairment, which allowed for the recognition of the needs of these people regarding the sounds with which they wish to become acquainted. The 35 initially proposed sounds were assessed using a five-grade scale. These sounds included those that would be heard in a number of situations in which a person with a visual impairment could potentially be found, both at home and, for example, while in the street or at an office. During the consultations, people with visual impairment usually rated the sounds proposed for inclusion in the set of sound exercises as highly relevant or relevant. In most cases, the assessment was analogous regardless of whether the person had a visual impairment since birth or developed it relatively recently. There were also more than 100 sounds that were proposed for inclusion in the set. The results of the consultation demonstrate how important the information contained in sound is for people with visual impairment.

## 1. Introduction

According to data published by the WHO in 2019, at least 2.2 billion people worldwide have a visual impairment or experience blindness [1]. However, the above data do not specify how many people are affected by total blindness. Previously published figures indicate that there are 39 million such people in the world [2]. In this regard, the WHO criterion for recognizing visual impairment as blindness is visual acuity worse than 3/60 (0.05 in decimal notation). These values represent the quotient of the distance from which a person reads signs during a test and the distance from which they should be able to see them with normal eyesight. Full visual acuity produces a test result of one. Data from the Central Statistical Office published in 2016 [3] indicate that in Poland, the number of people with visual impairment (damage and disease of the visual organ) in 2014, according to the statistical criterion, is 1,654,100. The statistical criterion covers individuals with legal disabilities and those who are not officially recognized as having a disability, but declare that they have limitations in carrying out normal activities (so-called biological disabilities).

It is well known that the reception of auditory stimuli plays a very important role in the functioning of people with visual impairment. Hearing is of paramount importance for the spatial orientation of blind people. Personal contact with people with visual impairment revealed that, due to the concerns these people have when faced with the prospect of leaving a well-known environment, such as their homes, it would be important for them to become familiar with situations, i.e., the surrounding sounds that they might face in an external environment. Therefore, the idea was to prepare a set of sound exercises (SSE) designed to develop the perception of sounds in the acoustic environment by people with visual impairment. This set should, among other things, enable people to listen to sounds that reflect real-life situations as closely as possible.

The results of auditory training sessions for blind and visually handicapped children and adolescents have shown that such training may be helpful, especially in tasks related to the localization of moving sound sources [4]. The suitability of training for the use of sound information in movement has also been confirmed in tests in which visual information is coded by means of spatial sound, using simulated echolocation and distance-dependent volume modulation [5]. In addition, using a virtual environment, it was found that it is possible to use navigation with sound-coded distance indication. This provided inputs that significantly improved the ability of blind people to cope with the real world [6]. The usefulness of sounds has also been confirmed in one experiment, in which information on a specific route was provided by sound effects on a step-by-step basis. Most of the 14 study participants were able to create an impression of the route, its structure, and characteristic points [7] based on the audible information received. In the case of blind people, information received in audible form may be sufficient enough to enable them to control the approach of an aircraft they are flying [8]. The simulations showed that maneuvering the aircraft solely on the basis of audible information enabled these people to guide the aircraft to the target with good accuracy. The sense of hearing may also be used to operate common-use equipment. Examples of such everyday equipment include a glucose meter equipped with a loudspeaker function for visually impaired and blind diabetes patients, a talking watch, or even a talking spirit level. Sound-equipped components are also included in more advanced solutions. An example of solutions that support the functioning of people with visual impairment is a virtual sense system, which is currently under development and is designed to support the navigation of blind and visually impaired people [9]. It is intended for indoor and outdoor use and does not require prior installation in the location of intended use. This system is intended to assist people in following walls and it features an acoustic compass as well as an algorithm for operation in a virtual acoustic space.

These examples show how the sense of hearing can play an important role in the functioning of people with visual impairment. These examples also provided encouragement for us to work on the above-mentioned SSE, which will facilitate the development of sound perception abilities. The first stage of SSE development was to determine the content of the set of sound signals and situations to be implemented. In order to determine the content of the set, people with visual impairment were consulted and we asked for their help in selecting the sounds. The aim of this work was to present the expectations of people with visual impairment regarding the content of a proposed set of sound exercises.

## 2. Materials and Methods

### 2.1. The Concept of the Set of Sound Exercises

The basic assumption made before developing the SSE was that it will enable people to become familiar with the sounds associated with certain situations and that it will train people in the perception of sounds, especially in terms of the recognition of the direction of a sound. The SSE will be used in equipment that enables the sound material to be played back over headphones connected, for instance, to a PC. The audio material will consist of recordings that will provide a spatial sound effect. In order to preserve the spatial impression associated with the correct direction of the sound, the sounds included in the set of exercises should be properly recorded, e.g., using the Ambisonics technique [10,11].

It was also assumed that the SSE will include sounds associated with specific life situations. Listening to these sounds could prepare a visually impaired person before they leave the home and familiar surroundings. Becoming familiar with sounds that can be found in real conditions will enable a visually impaired individual to recognize situations that require certain behaviors, such as the sound of an emergency vehicle. Using a set of exercises could enable people with visual impairment to adapt faster to their workplaces and achieve better spatial orientation at the workplace. However, the effects of using a set of exercises could be more extensive. Becoming familiar with the sounds of their surroundings, which these people do not normally experience (or are not fully aware of the information they contain), would enable them to become more independent or improve their quality of life. It is therefore also likely that, for example, it would increase their motivation to find a job.

The SSE planned for implementation will be based on specific recordings of sounds that can be found in everyday life. Hence, the presented consultations with people with visual impairment are a stage of the work that precedes the preparation of the SSE. The consultations are to enable the project team to decide which sounds should be included in the SSE. It is planned that the participant will have the possibility to limit the scope of the exercises to those groups of sounds that they find interesting. As already mentioned, it is also planned that this SSE will be implemented in the form of software to be run on a computer and will consist of two functional modules. In the first module, users using SSE-implementing software will learn sound by sound with a number of situations that may occur in everyday life. Auscultation with sounds will be supplemented with tests verifying the participants’ skills. As part of the test, participants will be asked questions about the sounds they have heard. After hearing each of the questions, the participants will be required to select the correct sound out of several suggested answers to describe a properly heard audio example. The test will check to what extent the person is able to recognize what the reproduced sounds are, based on the sound material they have already heard, while, at the same time, deepening their skills. The test results will be displayed to the participant, while, at the same time, being used to monitor their learning progress. The second SSE module will contain an exercise that teaches the participant to recognize the direction from which the sound is coming from. First, the participant will be presented with examples of sounds emitted by a specific source, registered in different directions towards the observer, and, in each case, the participant will be informed from which direction the sound is coming. Later, an exercise will be carried out in which the participant will not be informed of which direction the sound is coming from, but will have to guess this on their own. It is planned that this element of the SSE will be carried out with the use of various sound sources, e.g., the sound of a passing car. After a participant has completed a series of exercises, their exercise result will be displayed and they can compare this to the next exercise.

### 2.2. Participants in the Consultations

Consultations aimed at defining the content of the sounds to be included in the SSE were carried out with the participation of 20 people with visual impairment. Among them were blind people or individuals with a significant degree of impairment, aged between 23 and 49. The people consulted included 5 women and 5 men who had had a visual impairment since birth (or had developed one relatively long ago), as well as 5 women and 5 men who had developed an impairment relatively recently. The majority of these people, i.e., 18 of the 20, were professionally active. The other two individuals had also worked in the past.

### 2.3. The Proposed Sounds

**Figure 1:**
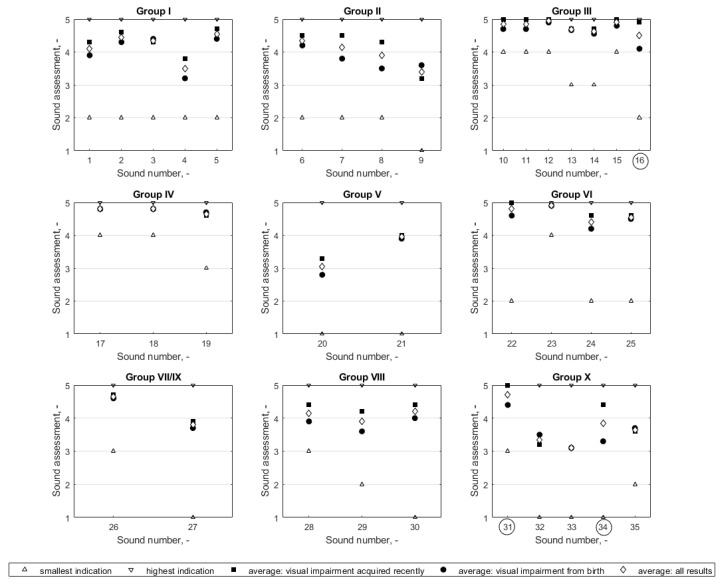
Evaluation of the proposed sounds. Sound numbers written inside the circles (16, 31, and 34) indicate cases when the differences between the average results of people who had a visual impairment since birth and who had developed an impairment relatively recently are statistically significant. Sound number designations: 1—an object falling onto a floor covered and not covered by carpet, 2—water dripping from a tap or shower head that is not completely off, 3—ring signal from a door entry phone, 4—conversations or sounds, e.g., from a radio heard through a wall, 5—knocking on a door, 6—steps on a staircase (sound reflections), 7—dog barking, 8—closing a gate, 9—low-flying passenger aircraft, 10—being surrounded by moving single-track vehicles (e.g., a scooter), 11—surrounded by moving two-track vehicles (e.g., passenger cars, trucks), 12—in the vicinity of moving rail vehicles (e.g., tram), 13—when staying on the pavement near a street with heavy traffic, at different distances from the street, e.g., 5 m, 10 m, 14—list of sounds present in the vicinity of streets with different traffic intensities, 15—signaling device at a pedestrian crossing, 16—bicycle bell, 17—a public bus pulling up to a stop, 18—the sound of a tram as it comes to a stop, 19—closing door of a public transport bus/tram, 20—engine operation, various types of vehicles, distinguishing between gasoline or diesel engines, 21—opening and closing a vehicle (car) door, 22—clapper sound, 23—a tram bell, 24—a signal to warn that a vehicle is driving backwards, 25—emergency vehicle signals, 26—vertical communication (passenger lifts), 27—reverberation in a building, 28—a xerograph or printer calibration suddenly starting, 29—a document being printed, 30—telephone ringing, 31—sound of a cyclist/person on rollerblades approaching on a cycling path, 32—bird sounds, 33—wind noise in the leaves of trees, 34—lawnmower powered by an internal combustion engine, 35—sounds coming from a playground for children.

For the purpose of the consultation, a preliminary proposal for the content of the sound set to be included in the SSE was made. The proposed sounds (or situations) were divided into 9 groups and are listed in Table 1. A detailed list of the sounds used is shown in the description under Figure 1.

Initial knowledge about the expectations of people with visual impairment was based on the authors’ personal contacts with people with disabilities, which took place during the implementation of projects at the Central Institute for Labour Protection-National Research Institute. This included, among others, a project entitled “Framework Guidelines for the design of facilities and adaptation of workstations for people with disabilities and specific needs” [12]. Recognition of the needs of people with visual impairment was also carried out in the form of work aimed at developing a test stand for research on sound perception with the participation of people with visual impairment [13]. In interviews with people with visual impairment, the most interesting situations, according to the interviewees, and the sounds associated with them were established. Personal contacts with people with visual impairment confirmed that the information contained in the acoustic signal is very important to them. The conclusions drawn from the statements of these people were used when preparing the proposal of 35 sounds for the consultation. The focus was on situations in which sound can provide important information for people with visual impairment. These people expressed the opinion that, for them, sound is an important source of information about their surroundings and they must try to use it, even in difficult acoustic conditions, i.e., when there are strong sound reflections (e.g., in reverberation rooms).

### 2.4. Assessment of the Proposed Sounds and Suggestions for Modification of the Proposed Set

Each of the participants of the consultation was asked to provide an opinion on what they expected in terms of the content of the set of sounds to be implemented in the SSE. An assessment of each of the items in the proposed sound set was requested. The assessment of each of these items involved answering the question, “Do you think this sound/situation is relevant (important, necessary) enough to be included in the SSE?” The assessment of each of the items from the list was requested using a five-grade scale: 1—completely irrelevant, 2—not very relevant, 3—neither irrelevant nor relevant (neutral grade), 4—relevant, 5—highly relevant. The participants were also asked to provide their own proposed sounds that could be added to the SSE.

### 2.5. Statistical Analysis

As mentioned above, the participants of the consultation were both people who had had a visual impairment since birth and those who had developed one relatively recently. A statistical analysis of the data obtained was carried out in order to assess whether there were significant differences in the assessment of the ratings of the individual sounds proposed, obtained from the two previously mentioned groups of people. Data were analyzed using MATLAB R2020b (version 9.9) software with the Statistics and Machine Learning Toolbox (MathWorks Inc., Natick, MA, USA). The analysis used the Wilcoxon test, equivalent to the Mann-Whitney U test.

## 3. Results

### 3.1. Assessment of the Proposed Sounds

The assessment of the individual sounds proposed for inclusion in the SSE was presented in the form of diagrams in Figure 1. The average values of the assessment ratings given were, in most circumstances, high, i.e., the sounds were evaluated as relevant or very relevant. In most cases, i.e., in 30 of 35 evaluated sounds/situations, the average ratings given by people who had developed visual impairment recently were higher than or equal to the average ratings given by people with visual impairment since birth, who were, therefore, more experienced and accustomed to the impairment. Only in a few cases, i.e., in 5 out of 35 of the evaluated sounds, did the opposite occur, i.e., people with visual impairment since birth rated a certain sound higher than those who had developed a visual impairment recently. It should be noted, however, that, in most cases, the differences in the average ratings between the two groups were not large (tenths of a point), reaching a maximum of 1.1 points for a five-grade assessment scale. The mean results and standard deviation values of the assessment of the proposed sounds are presented in Table 2 with a breakdown into groups of people, i.e., people who had developed visual impairment recently, people with visual impairment since birth, and all persons participating in the consultations. The Wilcoxon test indicated that the difference between the results of the assessment made by each of the two groups of people taking part in the consultations (with a visual impairment acquired recently and from birth) should be regarded as statistically significant only for 3 out of 35 of the sounds concerned. This was the case for a bicycle bell sound evaluated in Group III (‘p’ value was 0.0497) and for two sounds in Group X: the sound of cyclist/person on rollerblades approaching on a cycling path (*p* = 0.0143), and a lawnmower powered by a combustion engine (*p* = 0.0483). Each of these three sounds was rated higher by people who had developed a visual impairment recently. The differences between the two groups in these three cases were 0.8, 0.6, and 1.1, respectively.

### 3.2. Suggestions for Other Sounds in the Proposed Groups

The participants in the consultations submitted suggestions to be added to all the groups included in the set of sounds, except for Group VI (warning signals/audible emergency signals). Some of the suggested sounds were proposed not by one person but by two, and sometimes by several people. The participants jointly suggested 21 additional sounds for Group I, 10 sounds for Group II, 6 sounds each for Groups III and IV, 8 sounds for Group V, 13 sounds for Groups VII/IX, 8 sounds for Group VIII, and 11 sounds for Group X.

The sounds proposed to supplement the individual groups are listed below.

Group I:

1. Alarm clock ringing;
2. Water boiling;
3. Whistling kettle;
4. Knocking of blinds, shutters;
5. Gas ignition on a stove, gas stove igniter;
6. Breaking of glass/porcelain plate (falling on a hard floor);
7. Doorbell;
8. Footsteps on a staircase or nearby house, e.g., on a driveway;
9. Milk boiling over;
10. Pouring liquid into a dish/container without it overflowing;
11. Operating household appliances, e.g., kettle, vacuum cleaner, washing machine (end of washing cycle sound), oven (end of baking sound), etc.;
12. Switching an iron on and off;
13. Closing and opening doors;
14. Gas stove not working properly (gas leaking);
15. Flooding of a flat (sound of water flooding a flat) and water pouring onto the floor;
16. A fire (sounds of items burning, sizzling);
17. Induction stove sound push-buttons;
18. Electric oven sounds (various baking modes: top, bottom, thermal circulation, etc.);
19. Switching radio/TV or lights on/off in residential premises;
20. Preparation of meals (slicing, stewing, or frying of meat, etc.);
21. Telephone ringing.

Group II:

22. Sound of a lift (heard while in a hall)/lift door closing;
23. Opening the lock of an entrance door/the sound of a key in a lock;
24. A vehicle (or vehicles) driving by;
25. Sound made by keys when searching for them, e.g., in a bag or backpack;
26. Cat meowing;
27. Sound of neighbors’ doors or garage doors being closed;
28. Sounds related to cleaning staff working in staircases in residential buildings (floor cleaning, etc.);
29. A low-flying helicopter/small plane;
30. Large machines making loud noises (combine harvester, lawnmower, garbage truck);
31. A vehicle entering a car park.

Group III:

32. Roadworks, e.g., repairing traffic/streetlights (it is important that they have a distinctive sound, as this is relevant for blind people);
33. An approaching kick scooter/electric scooter, electric monocycle or self-balancing scooter, etc.;
34. A bicycle, scooter, or fast scooter passing by;
35. The sound of pedestrians’ footsteps crossing a street;
36. Sounds in public transportation;
37. Operation of a pneumatic hammer

Group IV:

38. Sounds related to an underground/approaching train and sound signals in an underground system;
39. Sound of doors opening (bus, tram, underground);
40. Voice signals in a bus or tram: announcements of stations/information about route diversion;
41. External voice communication (e.g., which bus it is);
42. Sound of pressing the ‘on-demand stop’ button, the alarm button on a bus or train;
43. A train.

Group V:

44. Vehicle braking/brakes squeaking;
45. Reversing vehicles;
46. Scooter ring signal;
47. Tractor, road cleaning vehicle (road sweeper)/snowplow during the winter;
48. Vehicle boot lid;
49. Refueling at a petrol station;
50. Pumping up bicycle tires;
51. Motorcycle engine starting.

Group VII/IX:

52. Sounds of footsteps on stairs;
53. Escalator/audio information about an escalator;
54. Opening and closing of automatic and revolving doors;
55. Voice information in lifts stating the floor numbers;
56. Guiding lines on a floor;
57. Synthetic speech sounds (machines, devices);
58. Sounds of buttons in lifts;
59. Fire alarms;
60. Sound of footsteps in a room (the difference between sounds depending on whether it is an open space or near a wall, etc.);
61. Sounds to locate a confessional and determine whether a blind person can approach it;
62. Bells in a theatre;
63. Sounds of footsteps of other people;
64. Echoes depending on the type of facility—a closed hall, railway and bus stations or large-format shops, etc.

Group VIII:

65. Noise of an operating computer, switching a computer/laptop on and off;
66. Sounds associated with a social room, e.g., a microwave oven;
67. Coffee machine/kettle;
68. Sounds of stamps being put on documents;
69. Destruction of documents in a shredder;
70. Fax sound/sounds of other electronic messages;
71. Air conditioner noise;
72. Sound of typing on a computer keyboard.

Group X:

73. Sound of a jogger/approaching jogger;
74. People approaching from the opposite direction;
75. Conversations of people sitting on benches/people playing various instruments;
76. Sounds from a grocery shop or restaurant (to locate the entrance);
77. Typical sounds of water streams;
78. Rustling grass sounds;
79. Sounds of dangerous animals/sounds of forest animals;
80. Sounds associated with a gradual/sudden change in the weather (e.g., thunder, gale, or rain);
81. Leaf blower;
82. Leaf raking;
83. Petrol-powered chainsaw.

### 3.3. Proposed Additional Sounds

In addition to the above sound suggestions to be added to the preliminary proposed sound groups (Section 3.2), the participants also suggested some additional sounds. Some of the additional proposals refer to the evaluated sound groups. The additional sounds suggested were as follows:

- Sounds common in the countryside: singing birds, croaking frogs, agricultural machinery, and sounds coming from the road.
- Sounds characteristic of shops and shopping centers: cash register, self-service cash register, payment terminal, putting shopping trolleys away, cleaning devices, and conversations.
- Sounds at public offices: the collection of a queue number from a machine or voice information announcing the next number in the queue.
- Train/underground station sounds: approaching and departing trains, train warning signals (loud horn), doors opening or closing, and voice messages.
- Airport: the sound of a plane taking off/landing and voice messages.
- Nature sounds: rainfall, gusts of wind, or thunder.

## 4. Discussion

Interviews conducted with the participation of visually impaired young people, including their parents have been used in several studies in order to find out about their expectations regarding various other activities intended to support them [14]. In the case of our work, information about the expectations of the SSE’s potential recipients took the form of consultations. An evaluation of how relevant the suggested sounds would be (important; necessary) for the planned SSE, as well as the possibility of proposing additional sounds, was included in the consultation. The consultations confirmed that each person has their own experience and, therefore, their own needs. In 8 out of the 35 sounds assessed, the individual ratings were vastly different. One person rated a particular sound as very relevant, while another as completely irrelevant. Overall, however, the consultations did not produce a significant variation in the assessment of the sounds concerned. On average, sounds were most often rated as relevant or highly relevant, and only in a few cases as neutral. It has also been proven that, apart from accidental cases, the average assessment of the usefulness of sounds is the same whether or not the respondents have developed a visual impairment recently or at birth. The differences between the assessment results made by each of the two groups of participants should be considered as statistically significant only for 3 out of 35 of the evaluated sounds. Even then, the differences in the average ratings between the two groups amounted to, at most, 1.1 on a five-grade evaluation scale. In general, in view of the fact that the differences in the assessments made by people with visual impairment developed recently and at birth, in most cases, do not differ statistically significantly, the average results for all persons taking part in the consultations can be assessed.

The consultations provided guidelines on the expectations of people with visual impairment for the contents of the set of sounds, and also an indication of the elements worth considering in order to extend the initially proposed set of sounds. Those consulted mentioned 83 sounds that could be added to the proposed set. In addition, these people proposed more than 20 other sounds from situations or places other than those previously assessed. Among the expected sounds were basic suggestions such as the “closing and opening of doors”. The importance of such sounds was confirmed, for example, in studies where navigation systems for the blind, using audio-based virtual environments, were tested [15]. Through elements and objects in the virtual environment, such as walls, stairwells, doors, toilets, or elevators, users can discover and come to know their location.

It is possible to approach the scope covered by the SSE differently. The consultations confirmed that, just as each person has their own individual character, each person has individual needs. A large group of people provides a better chance of noticing more sounds that can be included in the SSE. On the other hand, too large a set of exercises would be impractical. Therefore, when creating the software for implementing the SSE, it will be necessary to select the sounds arbitrarily, while taking into account the comments and experiences resulting from the consultations. In addition, it is planned that a person with little interest in a specific group of sounds will be able to exclude them over the course of the exercises. The software implementing the SSE will, to some extent, be prepared to take into account the individual needs of a specific user. For example, you can imagine that one person will be more interested in the sounds of a busy street and another in the sounds that can be found in an office. Due to the necessity to define the content of the SSE, the number of people participating in the consultations was not a critical factor related to their conduct.

It should be noted, however, that the limitation related to this manuscript is limited to 20 people participating in the consultations (10 people who had had a visual impairment since birth and 10 people who had developed an impairment relatively recently). Despite the fact that the statistics would be more reliable with the participation of a larger number of people, the consultations made it possible to identify the needs of people with visual impairment. It was particularly important to indicate numerous sounds that, in the opinion of the persons participating in the consultations, are worth being familiarized with. The consultations were devoted to the initial identification of the needs of people with visual impairment, so 20 participants seemed to be sufficient for this purpose. We deliberately included people with a visual impairment from birth and people who had developed an impairment relatively recently, with an equal share of women and men. A larger number of people is planned at the stage of assessing the prepared SSE, which will be implemented by taking into account the comments presented by the persons participating in the consultations.

In the past, research work was carried out on the ability to train people in terms of their hearing skills [4]. People who participated in the auditory training after its completion achieved, on average, a better result by about six percentage points compared to those who did not participate in the training. This directly confirms that sound exercise can be of benefit, which motivated us to develop the SSE. The verification test (before and after auditory training) described in [4] consisted of the following tasks: pitch discrimination, pitch and timbre categorization, pitch memory, lateralization of a stationary sound source, lateralization of a moving sound source, and lateralization of two moving sound sources. The results obtained by the authors of the study presenting the auditory training [4] did not show a clear increase in the number of correct indications in the case of tasks including pitch discrimination, pitch and timbre categorization, and pitch memory. On the other hand, such training improved the ability to determine the location of moving sound sources. When planning the scope of the SSE, it was also decided to focus on recognizing the direction of a sound. It is also planned to implement in the SSE the possibility of auscultation with sounds, which was a need reported by people with visual impairment. Another conclusion from the discussed article [4] was that the results were the same in the case of younger (7–12 years old) and older people (14–19 years old) participating in acoustic training. This knowledge is valuable because it indirectly allows us to conclude that auditory training is worth undertaking at any age. The authors of this paper, therefore, hope that the SSE we plan to develop will be useful regardless of the age of its potential users.

Auditory training conducted in the past with children and adolescents consisted of psychoacoustic tasks and sound lateralization: timbre discrimination, pitch discrimination, pitch memory, loudness discrimination, signal-in-noise detection, and sound source lateralization [16]. The approach that was used when developing the concept of the planned SSE is different, because it does not include the use of psychoacoustic tasks, but will be based on sounds recorded in real conditions. The common element, however, is the exercise of recognizing the direction of a sound.

In [17], the influence of the type of sound recording and the method of its reproduction in spatial orientation training for visually impaired children was investigated. Test signals were recorded both by the dummy head (binaural method) and by a microphone matrix (ambisonics). For presentation signals, in the case of a binaural method, two types (open and close) of headphones were used. The sounds recorded by microphone matrices were presented by loudspeakers. Studies have shown that, in the case of spatial orientation training for visually impaired children, the binaural method of recording test signals and its presentation through headphones is better than using microphone matrices and loudspeakers. In the planned SSE, binaural recorded sounds will be used, while headphones will be used to play back recorded sounds during exercises.

The consultations we have already held have shown that people with visual impairment need hearing exercises to be available to them in the form of a dedicated tool. Some people expressed this as an additional comment to the consultations performed. The importance of the solution is highlighted by the following statement relating directly to the purpose of the planned SSE: “I believe that such exercises should form an integral part of mobility training and everyday life learning activities involving people with impaired vision and blind people. They could also serve as a stand-alone exercise for improving sensitivity to sounds and helping to interpret these sounds appropriately, which would translate into both greater independence and safety”.

Currently, there are many tools available to support the functioning of people with visual impairment when they leave their homes. An example is a system for transmitting directional information using a matrix of electrodes that stimulate different parts of the tongue [2]. Another example of a system supporting visually impaired people is a solution supporting the spatial orientation of blind people using an ontology-based map of objects [18]. In this solution, similar to our work, sounds were also classified and recorded. Another example for supporting people with visual impairment when they leave their home is a system that, using a special electronic module, informs the driver that there is a person at a bus stop who is waiting for the arrival of a specific bus line [19]. The possibility of using SSE as a tool to prepare a user for a new environment, therefore, distinguishes the solution from the others, which provide support when a person is already in an environment outside their permanent place of residence.

## 5. Conclusions

During the consultations, people with visual impairment usually rated the sounds suggested for inclusion in the set of sound exercises as highly relevant or relevant. These sounds included a number of situations in which a person with a visual impairment could potentially be found, both at home and, for example, while in the street or at an office. In most cases, the assessment was analogous regardless of whether the person had a visual impairment since birth or developed it relatively recently. Those consulted also mentioned more than 100 sounds that could be added to the initially proposed sounds, amounting to 35 items. The results of the consultations held with people with visual impairment are a testimony to the great importance of the information contained in sounds for these people. The possibility of becoming familiar with these sounds and undergoing training to help with their recognition was appreciated. A solution of this kind is desired by people with visual impairment.

## Figures and Tables

**Table 1:** Groups of pre-proposed sounds for consultation with people with visual impairment.

Group Number	Group Name	Number of Sounds in the Group
I	Typical sounds heard inside the home	5
II	Situations outside the home	4
III	Traffic situations, being on the street and near junctions	7
IV	Typical sounds at places for the arrival/departure of means of transport	3
V	Sounds emitted by vehicles	2
VI	Auditory danger signals/audible emergency signals	4
VII/IX	Moving around within large buildings/in large premises (e.g., religious sites)	2
VIII	Sounds associated with office work	3
X	Typical sounds in recreational areas (e.g., gardens, parks or forests)	5

**Table 2:** Mean results and standard deviation values of the assessment of the proposed sounds.

Sound	Visual Impairment Acquired Recently		Visual Impairment from Birth		All Results	
	Mean	Std ^1^	Mean	Std	Mean	Std
1—an object falling onto a floor covered and not covered by carpet	4.3	1.1	3.9	1.0	4.1	1.0
2—water dripping from a tap or shower head that is not completely off	4.6	0.5	4.3	0.9	4.5	0.8
3—ring signal from a door entry phone	4.3	0.8	4.4	1.0	4.4	0.9
4—conversations or sounds, e.g., from a radio heard through a wall	3.8	1.2	3.2	0.6	3.5	1.0
5—knocking on a door	4.7	0.7	4.4	1.0	4.6	0.8
6—steps on a staircase (sound reflections)	4.5	0.8	4.2	1.1	4.4	1.0
7—dog barking	4.5	0.7	3.8	1.1	4.2	1.0
8—closing a gate	4.3	0.8	3.5	1.1	3.9	1.0
9—low-flying passenger aircraft	3.2	1.1	3.6	1.3	3.4	1.2
10—being surrounded by moving single-track vehicles (e.g., a scooter)	5.0	0.0	4.7	0.5	4.9	0.4
11—surrounded by moving two-track vehicles (e.g., passenger cars, trucks)	5.0	0.0	4.7	0.5	4.9	0.4
12—in the vicinity of moving rail vehicles (e.g., tram)	5.0	0.0	4.9	0.3	5.0	0.2
13—when staying on the pavement near a street with heavy traffic, at different distances from the street, e.g., 5 m, 10 m	4.7	0.5	4.7	0.7	4.7	0.6
14—list of sounds present in the vicinity of streets with different traffic intensities	4.7	0.5	4.6	0.9	4.6	0.7
15—signaling device at a pedestrian crossing	5.0	0.0	4.8	0.4	4.9	0.3
16—bicycle bell ^2^	4.9	0.3	4.1	1.1	4.5	0.9
17—a public bus pulling up to a stop	4.8	0.4	4.8	0.4	4.8	0.4
18—the sound of a tram as it comes to a stop	4.8	0.4	4.8	0.4	4.8	0.4
19—closing door of a public transport bus/tram	4.6	0.7	4.7	0.7	4.7	0.7
20—engine operation, various types of vehicles, distinguishing between gasoline or diesel engines	3.3	1.2	2.8	1.2	3.1	1.2
21—opening and closing a vehicle (car) door	4.0	0.7	3.9	1.6	4.0	1.2
22—clapper sound	5.0	0.0	4.6	1.0	4.8	0.7
23—a tram bell	4.9	0.3	4.9	0.3	4.9	0.3
24—a signal to warn that a vehicle is driving backward	4.6	0.7	4.2	1.0	4.4	0.9
25—emergency vehicle signals	4.6	0.7	4.5	1.0	4.6	0.8
26—vertical communication (passenger lifts)	4.7	0.5	4.6	0.7	4.7	0.6
27—reverberation in a building	3.9	1.1	3.7	1.3	3.8	1.2
28—a xerograph or printer calibration suddenly starting	4.4	0.5	3.9	0.6	4.2	0.6
29—a document being printed	4.2	0.8	3.6	1.1	3.9	1.0
30—telephone ringing	4.4	0.7	4.0	1.3	4.2	1.1
31—sound of a cyclist/person on rollerblades approaching on a cycling path ^2^	5.0	0.0	4.4	0.7	4.7	0.6
32—bird sounds	3.2	0.8	3.5	1.3	3.4	1.0
33—wind noise in the leaves of trees	3.1	0.9	3.1	1.2	3.1	1.0
34—lawnmower powered by an internal combustion engine ^2^	4.4	0.5	3.3	1.4	3.9	1.2
35—sounds coming from a playground for children	3.6	0.7	3.7	1.1	3.7	0.9

^1^ Std—standard deviation; ^2^ a comparison of the results of people who have developed a visual impairment recently and people with a visual impairment since birth showed statistically significant differences (*p* < 0.05).

## Data Availability

No new data were created or analyzed in this study.
